# Antimicrobial activity of customary medicinal plants of the Yaegl Aboriginal community of northern New South Wales, Australia: a preliminary study

**DOI:** 10.1186/s13104-015-1258-x

**Published:** 2015-06-30

**Authors:** Joanne Packer, Tarannum Naz, Yaegl Community Elders, David Harrington, Joanne F Jamie, Subramanyam R Vemulpad

**Affiliations:** Indigenous Bioresources Research Group, Faculty of Science and Engineering, Macquarie University, North Ryde, Sydney, 2109 Australia; Yaegl Local Aboriginal Land Council, Jubilee St, Hillcrest, Maclean, NSW 2463 Australia

**Keywords:** Antimicrobials, Multidrug resistant, Wound healing, Traditional medicine, Ethnobotany

## Abstract

**Background:**

This study is a collaboration between Macquarie University researchers and the Yaegl Aboriginal Community of northern NSW, Australia to investigate the antimicrobial potential of plants used in the topical treatment of wounds, sores and skin infections. Based on previously documented medicinal applications, aqueous and aqueous ethanolic extracts of *Alocasia brisbanensis*, *Canavalia rosea*, *Corymbia intermedia*, *Hibbertia scandens*, *Ipomoea brasiliensis*, *Lophostemon suaveolens* and *Syncarpia glomulifera* and the aqueous extracts of *Smilax australis* and *Smilax glyciphylla* were tested against common wound pathogens, including antibiotic resistant bacterial strains.

**Methods:**

Plant material was prepared as aqueous extractions modelled on customary preparations and using 80% aqueous ethanol. Extracts were assayed against a selection of clinically relevant Gram positive (*Streptococcus pyogenes* and sensitive and resistant strains of *Staphylococcus aureus*) and Gram negative (*Pseudomonas aeruginosa*, *Escherichia coli* and *Salmonella typhimurium*) bacteria and a fungus (*Candida albicans*) using disc diffusion and MTT microdilution methods. Viability of treated microorganisms was determined by subculturing from microdilution assays.

**Results:**

The extracts of *Corymbia intermedia*, *Lophostemon suaveolens* and *Syncarpia glomulifera* had promising levels of antimicrobial activity (MIC 31–1,000 µg/mL) against both antibiotic sensitive and resistant *Staphylococcus aureus* as well as the fungus *Candida albicans* (clinical isolate).

**Conclusion:**

Aqueous and 80% aqueous ethanolic extracts of *Lophostemon suaveolens*, *Corymbia intermedia* and *Syncarpia glomulifera* exhibited promising levels of antimicrobial activity against a range of both antibiotic sensitive and resistant strains of microorganisms. This is the first report of antimicrobial activities for *C. intermedia* and *L. suaveolens* and the leaves of *S. glomulifera*. This study demonstrates the value of customary knowledge in the identification of new sources of antimicrobial treatments.

**Electronic supplementary material:**

The online version of this article (doi:10.1186/s13104-015-1258-x) contains supplementary material, which is available to authorized users.

## Background

Australian Aboriginal people have over 40,000 years of knowledge of flora and fauna as sources of food, healing agents and other resources. Studies of customary (traditional and contemporary) medicinal plant preparations, especially in recent years, have revealed interesting medicinal properties and valuable biologically active compounds [1]. Australian Aboriginal medicinal flora have had limited biological screening studies aligned with their medicinal uses. Furthermore, the knowledge of their medicinal uses is quickly disappearing [2], particularly in some regions of the country, such as the southern states of Australia. Assessment of the bioactive potential of these plants with long historical use may support their wider application and also provide a source for safe, accessible alternative medicines and leads for the discovery of new drug-like molecules [3].

The ongoing crisis of antimicrobial resistance [4] calls for the investigation of novel sources of antimicrobials. Working with Indigenous communities who have been using natural remedies over the centuries can provide novel sources of antimicrobial therapies and/or lead compounds for the development of new medicines [3].

Discussions with Elders of the Yaegl Aboriginal community of New South Wales, Australia on their customary (traditional and contemporary medicines) identified plants used in the topical treatment of conditions of a microbial aetiology [5]. These included *Alocasia brisbanensis* for burns and boils, cuts, sores and open wounds; *Lophostemon suaveolens*, *Smilax australis*, *Smilax glyciphylla* and *Syncarpia glomulifera* for antiseptic purposes; *Canavalia rosea* for boils and sores, *Corymbia intermedia* for the treatment of wounds; *Hibbertia scandens* for sores and rashes; and *Ipomoea brasiliensis* as a poultice for boils.

There has been little investigation into these plants identified by the Yaegl community for their antimicrobial potential. This study looks into the antimicrobial activity of these plants against eight pathogenic organisms commonly implicated in wounds, sores and skin infections.

## Methods

### Ethics protocol

This research was approved by the Human Ethics Committee at Macquarie University, (HE27 JUL2007-R05356 and 5201200763). It was conducted under the framework of best ethical practice, working in partnerships with Indigenous people [6, 7] and was governed by a cooperative research agreement with the Yaegl community [8]. The choice of plants and the biological testing to be undertaken were determined in consultation with the Yaegl community through the Yaegl Local Aboriginal Land Council as the appropriate authorising body. The proposed methods and workflow were discussed before work commenced, and the results of the screening were presented back to the Yaegl community in the form of regular reports and presentations. The regular contact with the community during all phases of this research project ensured that the progress of the project remained in accordance with the requirements of the community and academics.

## Plant collection and identification

Plants were collected in March 2011 after being located and identified with the assistance of the knowledge holders of the Yaegl community. Plant identification was confirmed by and voucher specimens deposited at the Herbarium of the Royal Botanic Gardens of NSW or the Macquarie University Herbarium [registered with the Index Herbariorum, New York Botanic Gardens (http://sciweb.nybg.org/science2/IndexHerbariorum.asp)], with the prefix MQU.

For all plants the material used was leaves, except for *I. brasiliensis* where the stem and leaves were used. Plant material was weighed and washed twice with water to remove surface contaminants and extraneous material. Approximately 50 g of fresh plant material for each extraction method was collected (except for *S. australis* and *S. glyciphylla*, where 10 g was collected for the aqueous extraction only, due to inadequate availability of the plant material in the area).

## Chemicals

Unless otherwise indicated, all chemicals were sourced from Sigma Aldrich, St Louis, USA. All media listed below were sourced from Bacto Laboratories Pty Ltd, Australia and prepared as per the manufacturer’s instructions.

## Extraction of plant material

### Aqueous extraction of samples for antimicrobial screening

Plant material was, where possible, prepared in a way consistent with the preparation methods described by the Yaegl Elders [5] within 48 h of collection. In the case of pink bloodwood (*C. intermedia*) and apple gum (*L. suaveolens* or *S. glomulifera*), sap or latex while customarily used. Due to the risk of considerable damage to the tree, sap or latex was not collected and the Elders requested the leaves be collected and tested as an accessible and sustainable option. For *C. rosea* and *H. scandens,* the specifics of the preparation method could not be recalled by the knowledge holders.

Aqueous extractions were prepared as either a decoction or an infusion as described below.

Decoction (D): Plant material was pounded and boiled in 10× (*w/v*) tap water for 10 min, before being left to steep and cool (covered, for approximately 60 min). The decoction was filtered and the filtrate collected.

Hot infusion (I): Freshly boiled water was poured over roughly chopped or torn plant material (10× *w/v*). The infusion was covered and allowed to steep for 2 h. The infusion was filtered and the filtrate collected.

Cold infusion (CI): Plant material was roughly chopped or torn and submerged in 10× (*w/v*) water at room temperature. The infusion was shaken gently for 24 h, the mixture was vacuum filtered and the solid was shaken with fresh water (10× *w/v*) for an additional 24 h and filtered. The filtrates were pooled.

All crude aqueous extracts were dried by rotary evaporation (Büchi, Germany) at reduced pressure at approximately 50°C, followed by freeze drying (Labconco freeze dryer, USA). Extracts were stored at −20°C.

### Aqueous ethanolic extraction of samples for antimicrobial screening

Fresh plant material was ground to a coarse powder using a coffee grinder (IKA^®^ A11 Basic, Selangor, Malaysia) and shaken in 10× *w/v* of 80% *v/v* aqueous ethanol at room temperature [9] for approximately 48 h. The mixture was filtered and the solid shaken with fresh 80% *v/v* aqueous ethanol (10× *w/v*) for an additional 48 h, and the filtrates pooled. The ethanolic portion was removed by rotary evaporation under reduced pressure at a low heat (<40°C), leaving the water portion, which was freeze dried and stored at −20°C.

## Microbial strains

All extracts were initially screened against one Gram positive (*Staphylococcus aureus* ATCC 29213) and two Gram negative (*Escherichia coliβ*-lactamase negative ATCC 25922 and *Pseudomonas aeruginosa* ATCC 27853) bacterial strains, representing common pathogens associated with wounds, sores and skin infections [10, 11]. Selected extracts were also assayed against a broader range of microorganisms including: *Streptococcus pyogenes*—Group A, clinical isolate; *Salmonella typhimurium*—Group B, clinical isolate; resistant *S. aureus* strains (ATCC BAA 1026 community acquired methicillin resistant (CMRSA); wild multidrug resistant MRSA clinical isolate) and a yeast (*Candida albicans*—clinical isolate).

All microbial strains were kindly provided by Dr John Merlino (Department of Microbiology, Concord Hospital, Sydney) and the work was approved by the Macquarie University Biosafety Committee (Approval Reference 08/06/LAB, JOP250512BHA, TAN180512BHA). Müller Hinton II (MH) broth was used as the liquid medium for all bacteria; MH agar was used for the growth of *E. coli*, *S. aureus*, *S. typhimurium* and *P. aeruginosa* strains; horse blood agar (Edwards Instruments, Australia), was used for *S. pyogenes*. Potato dextrose agar (PDA, Oxoid, Basingstoke, UK) and Difco Sabouraud dextrose broth (SAB) were used for *C. albicans*. Gentamycin was used as the antibiotic control for the sensitive strains of *S. aureus*, *E. coli*, *S. typhimurium* and *P. aeruginosa*, vancomycin for the resistant strains of *S. aureus* and penicillin G for *S. pyogenes* (all antibiotics were supplied by Amresco, Ohio, USA). Fluconazole (MP Biomedicals, Ohio, USA) or miconazole (MP Biomedicals, LLC, France) was used as the antifungal control for the assays involving *C. albicans*.

## Disc diffusion assay

Dried plant material was dissolved in DMSO at 50 mg/mL. Antibiotic controls were prepared in broth at a concentration of 0.1 mg/mL. Sterile paper discs (∅ 6 mm, Whatman, Maidstone, UK) were impregnated with 2 × 10 µL of plant extract (1 mg/disc) or antibiotic control (2 µg/disc) and air-dried. 20 µL DMSO impregnated discs were included as a negative control. Discs were placed on appropriate agar plates (horse blood agar for *S. pyogenes,* potato dextrose agar for *C. albicans* or MH agar for other microorganisms), inoculated with broth cultures at A_600_ = 0.08 (bacteria: 10^7^–10^8^ cfu/mL, *C. albicans*: 10^5^ cfu/mL) and incubated at 37°C for 18–24 h (30°C for 48 h for *C. albicans*). The antimicrobial activity was measured as the zone of inhibition from the edge of the disc. Each sample and microbial combination was tested as at least four replicates.

## MTT microdilution assay

The assay was performed as outlined by Steenkamp et al., with minor modifications [12]. Plant extracts were prepared at 10 mg/mL in MH broth/20% DMSO (or SAB/20% DMSO for *C. albicans*) and serially diluted to give 20 μL final plant sample concentrations of 2–1,000 µg/mL (0.02–10 µg/mL of antibiotic control) in 96-well clear bottom microtitre plates. Test samples were inoculated with 175 µL of microbial culture (A_600_ = 0.08 diluted 1/100 in MH broth); a sterile broth control was included. The plates were incubated at 37°C for 18 h (30°C for *C. albicans*), 5 µL of a sterile methanolic solution (5 mg/mL) of MTT was added to each well and incubated at 37°C for 60 min. The MIC was the lowest concentration of test material in which no growth (as measured by no blue colour) was observed [13].

## Microbial viability determination

Plant extracts with antimicrobial activity (Table 1), as determined by the MTT microdilution assay, were assessed for their microbicidal/static nature by subculturing onto fresh agar plates after the assay [14]. Subcultures not producing colonies were deemed to have been affected by the plant extract by a microbicidal mechanism. The minimum microbicidal concentration was defined as the concentration of the plant extract in the last well showing no further microbial growth on subculture.

**Table 1:** Initial screening for antimicrobial activity of extracts of plants used customarily to treat skin conditions

Plant^a^ (voucher number)	Extract^b^	*Pseudomonas aeruginosa* (ATCC 27853)		*Staphylococcus aureus* (ATCC 29213)	
		Disc diffusion^c^	MTT^d^	Disc diffusion^c^	MTT^d^
		Average (range)	MIC median (range)	Average (range)	MIC median (range)
Apple gum					
*Lophostemon suaveolens* (Sol. ex Gaertn.) Peter G. Wilson & J.T. Waterh (MQ 73008908)	H_2_O (I)	na	na	2 (1–3)	188 (125–250)
	EtOH	na	na	3 (2.5–3.5)	93 (63–125)
*Syncarpia glomulifera* subsp. *glomulifera* (Sm.) Nied. (MQ 73009066)	H_2_O (I)	5.5 (0–10)	na	6.5 (5–10)	300 (250–500)
	EtOH	na	na	3 (3–6)	63 (31–63)
Beach morning glory					
*Ipomoea brasiliensis* (L.) Sweet (MQ 73007958)^A^	H_2_O (D)	na	na	na	na
	EtOH	na	na	na	na
Bloodwood					
*Corymbia intermedia* (R.T. Baker) K.D. Hill & L.A.S. Johnson (NSW 792670)	H_2_O (I)	1 (1)	na	2 (1–3.5)	250 (250–500)
	EtOH	na	na	2.5 (2–4)	299 (174–500)
Coastal jackbean					
*Canavalia rosea* (Sw.) DC. (MQ 73008909)	H_2_O (D)	na	na	na	na
	EtOH	na	na	na	na
Cunjevoi					
*Alocasia brisbanensis* Domin (MQ 73008737)	H_2_O (D)	na	na	na	na
	EtOH	na	na	na	na
Sarsaparilla					
*Smilax australis* R.Br. (wide leaf) (NSW 792674)	H_2_O (I)	na	na	na	na
*Smilax glyciphylla* Sm. (narrow leaf) (NSW 792380)	H_2_O (I)	na	na	na	na
Yellowvine					
*Hibbertia scandens* (Willd.) Gilg (MQ 73008905)	H_2_O (D)	na	na	na	na
	EtOH	na	na	na	na
Antibiotic control (Gentamycin)		8 (8)	1.25	8 (8)	0.5

*na* no activity detected.

^a^Leaves of plants used in all cases except where indicated.

^b^Extraction method used: H_2_O—based on traditional preparation (D, decoction; I, infusion; CI, cold infusion), EtOH—80% aqueous ethanol.

^c^Values given are inhibition in mm from the edge of the disc; average (range) of 4 replicates.

^d^MIC noted as µg/mL, based on the last dilution of extract not to exhibit yellow–blue colour change in MTT assay; values given are median (range) of four replicates.

^A^Leaves and stems.

## Results

Aqueous or 80% aqueous ethanolic extracts from nine plants used by the Yaegl community were initially tested against the Gram positive and Gram negative antibiotic sensitive bacteria, *S. aureus* (ATCC 29213), *E. coli* (*β*-lactamase negative, ATCC 25922) and *P. aeruginosa* (ATCC 27853) using the disc diffusion and MTT assays. The plant extracts showed activity only against *S. aureus* (in both disc diffusion and MTT assays) and *P. aeruginosa* (in disc diffusion assay only). *L. suaveolens, C. intermedia* and *S. glomulifera* extracts had the highest levels of antimicrobial activity (Table 1).

Antimicrobial activity for mixtures or crude plant extracts of between 0.1 and 1 mg/mL have been suggested as being appropriate for further investigation [15]. *L. suaveolens, C. intermedia* and *S. glomulifera* crude plant extracts were therefore further investigated as they had MIC values ≤500 µg/mL (Table 1). These extracts were tested against a broader spectrum of microorganisms viz. *Candida albicans*, *Streptococcus pyogenes*, *Salmonella typhimurium* and resistant strains of *S. aureus*—multidrug resistant (MDRSA) and community acquired methicillin resistant (CMRSA). Table 2 (and Additional file 1) presents the activities of these extracts. Results for *S. typhimurium* are not included as no activity was observed with any of the three plants. The 80% aqueous ethanolic extract of *S. glomulifera* was the most active of the extracts, based on low MIC values (31 µg/mL) against the tested bacteria as well as *C. albicans*. The 80% aqueous ethanolic extracts of both *L. suaveolens* and *S. glomulifera* were microbicidal in their activity against the susceptible microorganisms.

**Table 2:** Broad spectrum antimicrobial screening of extracts of selected plants

Microbe^A^:		CA		CMRSA		MDRSA		SP	
Plant	Ext^B^	Disc diffusion^C^ Average (range)	MTT MIC^D^ Median (range)	Disc diffusion^C^ Average (range)	MTT MIC^D^ Median (range)	Disc diffusion^C^ Average (range)	MTT MIC^D^ Median (range)	Disc diffusion^C^ Average (range)	MTT MIC^D^ Median (range)
*Corymbia intermedia* (R.T. Baker) K.D. Hill & L.A.S. Johnson (NSW 792670)	H_2_O	6.5 (6–8)	500 (250–500)	3 (2.5–3)	500 (250–500)	3 (2–4)	500	na	na
	EtOH	2 (0–6.5)	500 (250–500)	2.5 (2–5.5)	375 (250–500)	2.5 (1.5–4)	250 (250–500)	na	250 (125–>1,000)
*Lophostemon suaveolens* (Sol. ex Gaertn.) Peter G. Wilson & J.T. Waterh (MQ 73008908)	H_2_O	6 (5.5–6)	1,000 (500–1,000)	2.5 (2–3)	1,000 (500–1,000)	2.5 (2–3)	1,000 (250–1,000)	na	na
	EtOH	6.5 (6–7)	125 (31–125)	3.5 (3–4.5)	63 (63–125)	3.5 (3–5)	125 (63–250)	na	125 (16–>1,000)
*Syncarpia glomulifera* subsp. glomulifera (Sm.) Nied. (MQ 73009066)	H_2_O	6.5 (5.5–7.5)	500 (250–1,000)	3 (3–4)	375 (250–500)	2.5 (2–3)	250 (31–500)	na	na
	EtOH	5.5 (5–6)	31 (16–1,000)	5 (2.5–5)	31 (16–1,000)	3.5 (2–5)	31 (16–>1,000)	3 (2–4.5)	31 (31–>1,000)
Antibiotic control		17^a^	2.8^b^	3^c^	1.5^c^	2^c^	1.6^c^	8^d^	0.02^d^

Antibiotic controls: ^a^fluconazole, ^b^miconazole ,^c^vancomycin, ^d^penicillin.

*na* no activity detected.

^A^CA: *Candida albicans* (clinical isolate); CMRSA: *S. aureus* (ATCC BAA 1026); MDRSA: *S. aureus* (multidrug resistant clinical isolate); SP: *Streptococcus pyogenes* (Group A—clinical isolate).

^B^Extraction method used: H_2_O, traditional prep; EtOH, 80% aqueous ethanol.

^**C**^Values given are inhibition in mm from the edge of the disc; average of four replicates (plant extracts tested at 1 mg/disc and antibiotic controls tested at 2 µg/disc).

^D^MIC noted as µg/mL, based on the last dilution of extract not to exhibit yellow–blue colour change in MTT assay; values given are median (range) of 4 replicates.

## Discussion

Ethnobotanical studies conducted by us with Yaegl Aboriginal Elders of northern New South Wales, Australia, described the use of *Alocasia brisbanensis*, *Canavalia rosea*, *Corymbia intermedia*, *Hibbertia scandens*, *Ipomoea brasiliensis*, *Lophostemon suaveolens*, *Smilax australis*, *Smilax glyciphylla* and *Syncarpia glomulifera* for the treatment of wounds, sores and skin infections [5]. None of these plants have been investigated for their antimicrobial activity, with the exception of *C. rosea* [16]. The success of finding antimicrobial activity and bioactive compounds of medicinal value from traditional/customary medicines [3], prompted the investigation of these plants.

The plants were extracted with water to mimic the common customary practice of the community and/or with 80% aqueous ethanol, as a standard natural products research method [9]. The plant parts extracted were the same as those used by the Yaegl community, except for bloodwood (*C. intermedia*) and apple gum (*L. suaveolens* or *S. glomulifera*), where customarily the sap or latex was used in the treatment of wounds. However, as sap or latex were not readily accessible, we tested the leaves at the request of the Yaegl Elders.

The microorganisms selected for the assays are commonly implicated in wounds, sores and skin infections [11]. The two assay methods were chosen as they complement each other in that the disc diffusion assay is simple but not always appropriate for non-polar and large molecules [17] and the more sensitive, versatile and quantitative MTT microdilution assay can have interference with the colour and redox activity of plant extracts.

The extracts of *C. intermedia*, *L. suaveolens* and *S. glomulifera* had reasonable levels of antimicrobial activity (MIC 0.1–1 mg/mL; [15]) against antibiotic sensitive and resistant *Staphylococcus aureus* as well as the fungus *Candida albicans* (clinical isolate). Although antibacterial activity of the ethanolic extract of *C. rosea* leaves has been previously reported against *S. aureus*, albeit at a low level [16], no antibacterial activity was seen in this study, possibly due to the different bacterial strains and assay conditions used.

The 80% aqueous ethanolic extracts of *C. intermedia*, *L. suaveolens* and *S. glomulifera* were substantially more active than the aqueous extracts. This indicates that the antimicrobial components in these plants were more easily extractable in 80% aqueous ethanol. *S. glomulifera* was the most active of the extracts (Table 2). The 80% aqueous ethanolic extracts of both *L. suaveolens* and *S. glomulifera* were microbicidal in their activity against the susceptible microorganisms.

Sap from bloodwood (*C. intermedia*) has been used by the Yaegl community in the treatment of wounds [5]. No chemical or biological studies have been reported for *C. intermedia.* Milky sap and ash from the bark of the apple gum (*Lophostemon suaveolens* or *Syncarpia glomulifera*) have been used as antiseptics by the Yaegl community [5]. These two plants are sometimes interchangeably used due to their similar morphology.

No antimicrobial activity studies have been reported for *L. suaveolens* or the leaves of *S. glomulifera*. However, both plants have been found to contain the following compounds that have been independently reported for antimicrobial activity [18–22]. Leaves of *L. suaveolens* have been found to contain α-pinene, β-caryophyllene, aromadendrene, globulol and spathulenol [23], which have been independently reported for antimicrobial activity. The leaves of *S. glomulifera* also contain α-pinene, aromadendrene and globulol [24]. Eucalyptin, which has been reported to be antibacterial, has been found in the leaf wax of *S. glomulifera* [25]. Additionally, *S. glomulifera* bark has been reported to be antibacterial and to contain the antibacterial compounds betulinic acid, oleanolic acid-3-acetate and ursolic acid-3-acetate [26].

This is the first report of antimicrobial activities for *C. intermedia* and *L. suaveolens* and the leaves of *S. glomulifera*. These results warrant further investigation of the active ingredients of these medicinal plants.

## Conclusion

80% aqueous ethanolic extracts of *Lophostemon suaveolens* and *Syncarpia glomulifera* showed microbicidal activity at concentrations of <200 µg/mL against *C. albicans* and both sensitive and resistant strains of *S. aureus*. Aqueous extracts of *L. suaveolens* and *S. glomulifera* and aqueous and ethanolic extracts of *Corymbia intermedia* exhibited microbicidal activity against a range of both antibiotic sensitive and resistant strains of microorganisms at levels below 1,000 µg/mL. This is the first report of antimicrobial activities for *C. intermedia* and *L. suaveolens* and the leaves of *S. glomulifera*. This study demonstrates the value of customary knowledge in the identification of new sources of antimicrobial treatments.

## Additional file

Additional file 1. Picture showing typical results of the disc diffusion assay.
