# Supplementary material for: Antimicrobial activity of customary medicinal plants of the Yaegl Aboriginal community of northern New South Wales, Australia: a preliminary study
Source: BMC Res Notes. 2015 Jun 30;8:276. doi: 10.1186/s13104-015-1258-x (PMC4485878; doi:10.1186/s13104-015-1258-x)

**Additional File 1**

Picture showing typical results of the disc diffusion assay. Agar plate showing inhibition of growth of *Staphylococcus aureus* (CMRSA) in the presence of discs containing plant extracts (1 – 6, see below) or antibiotic control (AB = vancomycin). D = Disc with 20 μL DMSO.

1& 2 *Syncarpia glomulifera* Water and Ethanol extracts respectively

3 & 4 *Corymbia intermedia* Water and Ethanol extracts respectively

5 & 6 *Lophostemon* *suaveolens* Water and Ethanol extracts respectively

(7 is a substance not related to this manuscript.)


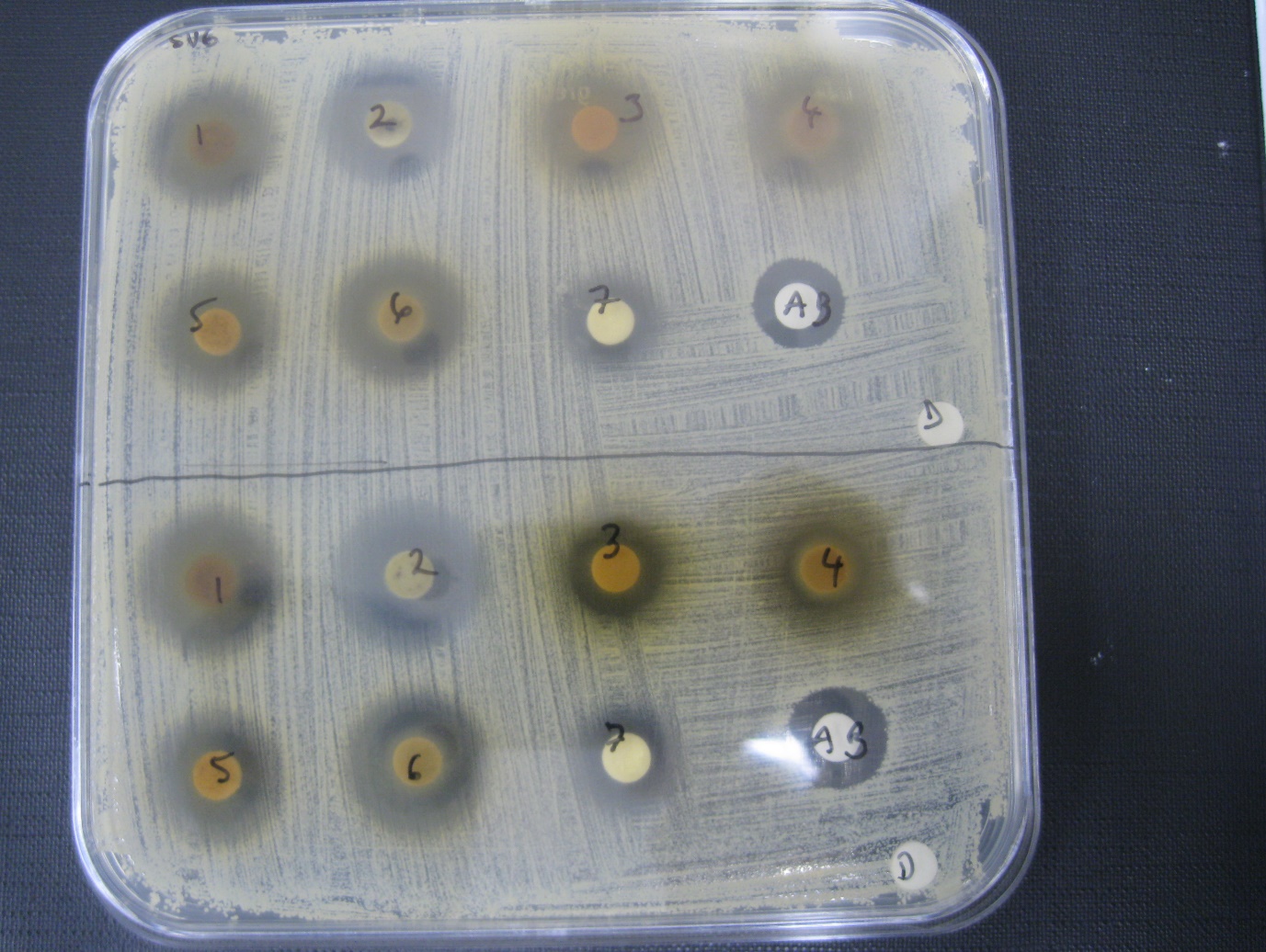

Supplement: Additional file 1. — Picture showing typical results of the disc diffusion assay. [file 13104_2015_1258_MOESM1_ESM.docx]
